# Supplementary material for: Effect of prenatal care quality on the risk of low birth weight, preterm birth and vertical transmission of HIV, syphilis, and hepatitis
Source: PLOS Glob Public Health. 2023 Mar 29;3(3):e0001716. doi: 10.1371/journal.pgph.0001716 (PMC10058091; doi:10.1371/journal.pgph.0001716)
Supplement: Attachment — Submitted filename: Response to Reviewers.docx [file pgph.0001716.s001.docx]

**Aguiar *et al*. "Effect of prenatal care quality on the risk of low birth weight, preterm birth and vertical transmission of HIV, syphilis, and hepatitis." (manuscript PGPH-D-22-00381)**

July 15^rd^ 2022, Rio Branco, Acre

Dear editor of the *PLOS Global Public Health*,

We are grateful for your e-mail dated July 6^st^ 2022 concerning the manuscript entitled “Effect of prenatal care quality on the risk of low birth weight, preterm birth and vertical transmission of HIV, syphilis, and hepatitis” (PGPH-D-22-00381).

We greatly appreciate all the contributions from the reviewers to improve the quality and clarity of our manuscript. We have carefully followed reviewers’ comments, and our point-by-point response is provided below, indicating how and where (line numbers) changes were proceeded. Changes are highlighted in red in the text.

Sincerely,

Débora Aguiar

(on behalf of all the co-authors)

Journal Requirements:

1. Please amend your detailed Financial Disclosure statement. This is published with the article. It must therefore be completed in full sentences and contain the exact wording you wish to be published.

**Response:** We totally comprehend the need of funding disclosure.

a. Please clarify all sources of funding (financial or material support) for your study. List the grants (with grant number) or organizations (with url) that supported your study, including funding received from your institution.

**Response:** This study was financed in part by Programa de Pesquisa Para o SUS-Fundação de Amparo à Pesquisa do Estado do Acre (PPSUS/FAPAC 2013 6068-14-0000032) and (PPSUS/FAPAC 2015 32888.501.20968.22032016).

This study was financed in part by the Coordenação de Aperfeiçoamento de Pessoal de Nível Superior-Brasil (CAPES)-Finance Code 001.

b. State the initials, alongside each funding source, of each author to receive each grant.

**Response:** Programa de Pesquisa Para o SUS-Fundação de Amparo à Pesquisa do Estado do Acre (PPSUS/FAPAC 2013 6068-14-0000032). RAMALHO, AA.

Programa de Pesquisa Para o SUS-Fundação de Amparo à Pesquisa do Estado do Acre (PPSUS/FAPAC 2015 32888.501.20968.22032016). ANDRADE, AM.

Coordenação de Aperfeiçoamento de Pessoal de Nível Superior-Brasil (CAPES)-Finance Code 001. AGUIAR, DM.

c. State what role the funders took in the study. If the funders had no role in your study, please state: “The funders had no role in study design, data collection and analysis, decision to publish, or preparation of the manuscript.”

**Response:** The funding agencies are Research funding institutions with academical purpose, only. Therefore, the funders had no role in study design, data collection and analysis, decision to publish, or preparation of the manuscript.

d. If any authors received a salary from any of your funders, please state which authors and which funders.

**Response:** We totally comprehend the need of funding disclosure. However, none of the authors received a salary from any funding supporters.

RESPONSE TO REVIEWER #1

Thanks for the work you've done. It's has important findings, especially for poor resource settings in a large country, like Brazil. Despite its importance, your work would benefit from some amendments in the text. Please, consider my comments below.

***Abstract***

Line 29: I think is obsolete and prejudiced the usage of the terms “developed and developing countries”. Please, consider my suggestion of replacing it by high-income and low and middle-income countries. Also, there’s a typo in the same line.

**Response:** We appreciate and agree with the suggestion. In this way, we modified the whole manuscript as follows High-income countries and Low-and-middle-income countries. Also, we corrected the typo as follow:

“Despite the advances made in maternal and child health, adverse birth outcomes still affect a significant portion of newborns in both, high-income and low and middle-income countries”.

(Please, see lines 33-35).

Line 31: Maybe I’m outdated, but the common term used is “postpartum women” instead of “puerperaes”. But this is out of my scope in this review.

**Response:** We agreed with the reviewer and checked the literature. The most updated term is “postpartum women”. Thus, we modified the term as suggested in the whole manuscript, as in the example below:

“Thus, this article to evaluate the effect of the quality of prenatal care process on birth indicators in a cohort of postpartum women who attended maternity hospitals in Brazilian western Amazon, city of Rio Branco, in the state of Acre, Brazil, in 2015”.

(Please, see lines 35-38).

Line 32: The Methods section needs some rearrangement. I would say that the definition of adequate PC is essential to understand the studied groups, instead of describing who is exposed or not. Covariates used to adjust the models were not described.

**Response:** We appreciate the suggestion and have rearranged the Method section as recommended (please, see lines 40-49). Therefore, the changes were proceeded as follow:

“This research was a hospital-based cohort study. The sample consisted of 1,030 women who gave birth in maternity hospitals in Rio Branco between April 6^th^. and June 30^th^., 2015. Prenatal care was classified as fully adequate when started ≤4th month; ≥80.0 – 109% to expected consultations for GA according to the Kotelchuck Index; ≥5 records of blood pressure, weight, GA, fundal height, ≥4 records of fetal heart rate, fetal movements or equivalent to 75% of the number of consultations; in addition to recording ABO/RH, hemoglobin, VDRL, urine, glucose, anti-HIV and anti-toxoplamosis during the 1st trimester. When all those criteria were met, the woman was classified in the level of adequacy (level-4).

Line 33: Same as the comment above, the Results section also needs some changes. I’d like to see the crude and adjusted figures for OR.

**Response:** We agreed with the reviewer, and include crude and adjusted OR: “Results: The incidences were 8.8% for LBW, 9.2% for preterm birth, 39.4% and 1.1% for vertical transmission (syphilis/HIV/hepatitis). Crude and adjusted OR showed that inadequate PC increased the risk of syphilis/HIV/hepatitis vertical transmission (ORcrude: 1.23; 95%CI: 0.26-5.71), LBW (ORcrude: 1.84; 95%CI: 0.99-3.44; ORadjusted: 1.87; 95%CI: 1.00–3.52), and preterm birth (ORcrude: 1.79; 95%CI: 1.00-3.29; ORadjusted: 3.98; 95%CI: 1.40–11.29)”

(Please, see lines 53-59).

***Introduction***
The comment about the terms “developed and developing countries” and “puerperae” applies to the whole text. **Response:** We appreciate the comment and revised the terms throughout the text.

Line 52: I don’t think that is plausible to compare mortality rates between Latin American countries, where progress has been reached in the last decades on reducing the burden of under 5-y mortality, with Bangladesh that still struggles to reduce the high mortality rates.

**Response:** We comprehend the reviewer’s suggestion, and proceeded the changes as follow: “Mortality of children under 5 years of age remains a major public health challenge in low- and middle-income countries, such as Colombia, Mexico, and Brazil”.

(Please, see the Introduction section, lines 64-66)

***Methods***
Please, provide details about the study site. Where is Rio Branco located, how many people live there, is it a big city, how many maternity wards exist in the city, among other basic characteristics?

**Response:** We recognize that the study location description is missing in the manuscript. Thus, we included a paragraph with such information, as follow:

“Rio Branco is located in the state of Acre, western Brazilian Amazon area, which comprises the greatest ethnic mixed population, and this region it has the greatest miscegenation and presence of indigenous people in the country^27^. Acre encompass around 906.876 inhabitants^28^. Rio Branco city holds only two maternity hospitals, one public and one private that also serves the SUS in a complementary way.”

(please, see the Methods section, lines 130 - 135).

REFERENCES:

27. Brasil. Instituto Brasileiro de Geografia e Estatística—IBGE. Censo Demográfico. IBGE: Características da População e dos Domicílios: Resultados do Universo. In IBGE; Sidra: Sistema IBGE de Recuperação Automática; Instituto Brasileiro de Geografia e Estatística—IBGE: Rio de Janeiro, Brasil, 2011. Available online: http://www.sidra.ibge.gov.br/cd/cd2010universo.asp?o=5&i=P (accessed on 21 Jul 2022).

28. Brasil. Instituto Brasileiro de Geografia e Estatística—IBGE. Censo Demográfico. IBGE: Cidades e Estados: Rio de Janeiro, Brasil, 2022. Available online: <https://www.ibge.gov.br/cidades-e-estados/ac/.html> (acessed on 21 Jul 2022).

Line 135: What’s the difference between “outcomes at childbirth and birth”?

**Response:** We appreciate the comment and recognized that there was a mistake in the translation process. Actually, we meant “***labor and childbirth outcomes***” instead of “outcomes at childbirth and birth”. Thus, we have modified the term throughout the manuscript.

Line 136: It’s important to provide the definitions used for each outcome.

**Response:** We appreciate the suggestion and add the following: Maternal and infant outcomes at labor and childbirth included LBW (babies who are born weighing less than 2,500 grams), preterm birth (birth of a baby at fewer than 37 weeks) and vertical transmission of HIV/hepatitis/syphilis (diagnosed by mother-and-child anti-HIV/hepatitis/syphilis serological tests).

(please, see the Methods section, lines 155 - 157).

Additionally, it would be great to see the details about the Kotelchuck index, because I think a bit though to keep reading the findings of your research without further details.

**Response:** We agree that Kotelchuck index information is missing. Therefore we have included such an information, as follow:

“The Kotelchuck index categories were calculated considering the gestational age (GA) at PN care onset, categorized as 1 and 2 months, 3 and 4 months, 5 and 6 months and 7 to 9 months, while the ratio of the number of consultations observed over the number of consultations was obtained by the ratio of the number of consultations observed to those expected for each GA. The final measure is obtained by combining these two dimensions and classifying them into: Adequate plus: beginning before or during the 4th month and 110.0% of consultations; Adequate: start before or during the 4th month and 80.0 to 109.0% of consultations; Intermediate: start before or during the 4th month and 50.0 to 79.0% of consultations and Inadequate: start after the 4th month and/or less than 50.0% of consultations expected for the gestational age”.

(please see the Methods section, lines 162 - 173).

It’s crucial to provide information about the covariates available and selected to perform the multivariate analysis.

**Response:** We recognize that descriptions regarding covariates available and selected are missing. Thus, we have included such descriptions as follow:

“Subsequently, the crude and adjusted odds ratios (OR) were estimated, with their respective 95% confidence intervals, between the exposure variables: Maternal age (<20 years; 20-34 years; ≥35 years), self-reported skin color (white; mixed-race; others), Education level (elementary school; high school; college degree), Marital status (no partner; with partner), Family income (up to 1 MW; 1-3 MW; >3 MW), ABEP class (A and B; C, D and E), Planned pregnancy (no; yes), Prenatal care (public setor; private sector), Abortion (no; yes), Number of childbirths (primiparous; multiparous) and the outcomes, using non-conditional logistic regression, with Wald statistical test. We proceeded multivariate analyses, for each association between PC quality care levels (Level-1, 2, 3, and 4) with each outcome (LBW and preterm birth), considering the same set of covariable for adjustment. Thus, multivariate analyzes were proceeded considering the following criteria for covariates inclusion in the model: statistical significance (p<0.20) in the crude OR analysis, biological plausibility in the causal process. The criteria to keep the covariates in the final model included a p-value ≤0.05, biological significance, and a confounding effect detected by modifying the OR magnitude over 10% after adjustment.”

(please see the Methods section, lines 202 - 217).

***Results***
Line 187: Usually, the first thing to describe in this section is the overall frequencies/prevalence of the exposures and outcomes. Table 1 contains this information by levels of exposure.

**Response:** We add the requested information “The highest incidence of LBW was found in women with inadequate PC in all levels of adequacy, equivalent to 9.7% at level-1, 9.0% at level-2, 9.9% at level-3 and 9.7% at level-4. The same result was found for preterm birth, with a higher incidence among women with intermediate/inadequate PC, equivalent to 9.7% at level-1, 11.5% at level-2, 9.9% at level-3 and 10.1% at level-4. The incidence of vertical transmission (syphilis/HIV/hepatitis) was also prevalent among women with intermediate/inadequate PC at level-1, level-2, level-3 and level-4, corresponding to 2.2%, 1.2%, 1.3% and 1.1%”.

(please see the Results section, lines 222 - 229).

Line 189: Findings for LBW were not statistically significant for any exposure level investigated. I think it does not worth mentioning in detail such findings as they are given in Table 1.

**Response:** We understand the reviewer’s comment, however we have considered that the crude magnitude of associations for both levels could suggest real associations between inadequate PC in level-3 (OR=1.62) and level-4 (OR=1.84). Although the 95% confidence intervals dis not show a statistical significance for level-3 (0.97 – 2.72) and level-4 (0.99 – 3.44), the 95%CI were borderline for both levels. Therefore, the lack of statistical significance probably occurred because our sample size did not provide enough statistical power to the detect an association that could be real. Thus, in this case, statistical significance only tells us about the likely of random error. If our sample size was greater, such an association would have been statistically significant.

Also, after adjusting by confounding variables, statistically significant associations were observed for both levels (please see table-3).

However, we recognize that the absence of statistical significance in the crude analyzes was not mentioned in the text. Therefore, we rewrote the text as follow:

“Despite a borderline 95% confidence interval, women classified with intermediate/inadequate PC were 62% more likely to have LBW child (95% CI: 0.97–2.72) than those with adequate plus/adequate PC, for level-3 criterion. Similarly, for level-4 criterion, women with intermediate/inadequate PC were 84% more likely to have LBW child (95% CI: 0.99–3.44) than women with adequate plus/adequate PC, despite the borderline 95% CI”.

(please see the Results section, lines 240 - 245).

Line 209: Table 2 should be the first table of your paper.

**Response:** We agreed with the reviewer. So, we have changed the table order, as suggested.

Line 217: I found confusing the description of adjusted models as different covariates were used in each model. Please, provide information in the Methods section of the covariates considered in the analysis. Did you select a different set of covariates for each level of exposure? I miss that part.

**Response:** We recognized that final models for each outcome, according to the PC quality care levels were not well explained. In fact, in the multivariate analyses, for each association between PC quality care levels (Level-1, 2, 3, and 4) with each outcome (LBW and preterm birth) we produced a different final model, using the same set of covariable. Thus, we included such information, as follow:

“We proceeded multivariate analyses, for each association between PC quality care levels (Level-1, 2, 3, and 4) with each outcome (LBW and preterm birth), considering the same set of covariable for adjustment. Thus, multivariate analyzes were proceeded considering the following criteria for covariates inclusion in the model: statistical significance (p<0.20) in the crude OR analysis, biological plausibility in the causal process. The criteria to keep the covariates in the final model included a p-value ≤0.05, biological significance, and a confounding effect detected by modifying the OR magnitude over 10% after adjustment”.

(please see the Methods section, lines 210 -217).

Table 3: It looks unfinished. The style is different from the other tables.

**Response:** We proceeded the multivariate analyses, for each association between PC quality care levels (Level-1, 2, 3, and 4) with each outcome we produced a different final model, using the same set of covariable according to the criteria established in the methods with each outcome (LBW and preterm birth). However, for facilitate the understanding of the results, we have included a dash to inform that in that model specifically that covariate and the outcome did not meet the criteria considered part of the model.

However, we agreed with the reviewer and changed the table style to facility the comprehension.

***Discussion***
Lin 245: This first paragraph looks like the reasons for conducting your study and would be better placed in the Introduction. T**he *first paragraph of the Discussion is a synthesis of the main findings of your research. Please, consider rewriting it***.

**Response:** We accepted the suggestions and modified “The results that the incidence of LBW in women with PC Adequate plus/adequate decreased from the moment the Kotelchuck index + laboratory procedures were considered (6.3%) or when all recommendations at Level-4 were considered (5.5%). Thus demonstrating the importance of all procedures, especially laboratory procedures to reduce conditions that cause LBW. Preterm birth had a lower incidence in women with Adequate plus/adequate prenatal care considered the Kotelchuck index + obstetric procedures (6.4%) and all procedures at Level-4 (5.9%). Demonstrating the importance of such procedures, especially obstetrics to reduce prematurity.

Vertical transmission (syphilis/HIV/hepatitis) had the lowest incidence among women with adequate plus/adequate prenatal care, considering the Kotelchuck + laboratory tests index (0.6) and only the Kotelchuck index (0.7%). Demonstrating the importance to early prenatal care, in addition to the minimum number of consultations to detect morbidities that can be treated with the aim of preventing vertical transmission.”

Overall: The authors seemed to focus on seeking other findings in the literature that corroborated with theirs, instead of deeply discussing your results in light of the challenges and strategies to overcome such barriers for optimal prenatal care, especially in poor-resource settings in Brazil. My recommendation is to review this section thoroughly.

Recently, a new analysis was published about the quality and content of prenatal care in low- and middle-income countries. I suggest you take a look at this work which might be useful.
https://jogh.org/documents/2021/jogh-11-04008.pdf
<https://equityhealthj.biomedcentral.com/articles/10.1186/s12939-021-01440-3>

**Response:** We appreciate the reviewer comment and suggestion. Therefore, we have revised and modified the discussion section focusing not only on findings in the literature that corroborated with ours, but also discussed our results in depth regarding the PC quality challenges in LMIC and in poor-resource settings in Brazil.

Also, we used the references suggested by the reviewer, as well as new ones.

REFERENCES:

Arsenault C, Jordan K, Lee D, Dinsa G, Manzi F, Marchant T, et al. Equity in antenatal

care quality: an analysis of 91 national household surveys. Lancet Glob Health.

2018;6:e1186-95. Medline:30322649 doi:10.1016/S2214-109X(18)30389-9.

Victora CG, Requejo JH, Barros AJ, et al. Countdown to 2015: a decade of tracking progress for maternal, newborn, and child survival. Lancet 2016; 387: 2049–59; Gomez GB, Kamb ML, Newman LM, Mark J, Broutet N, Hawkes SJ. Untreated maternal syphilis and adverse outcomes of pregnancy: a systematic review and meta-analysis. Bull World Health Organ 2013; 91: 217–26.

Arroyave L, Saad GE, Victora CG, Barros AJD. A new content-qualified antenatal care coverage indicator: Development and validation of a score using national health surveys in low- and middle-income countries. J Glob Health 2021;11:04008.

Arroyave, L., Saad, G.E., Victora, C.G. et al. Inequalities in antenatal care coverage and quality: an analysis from 63 low and middle-income countries using the ANCq content-qualified coverage indicator. Int J Equity Health 20, 102 (2021). https://doi.org/10.1186/s12939-021-01440-3.

RESPONSE TO REVIEWER #2

Dear editor, thanks for the opportunity to review this manuscript.
***Methods***
1) Variables that were considered for adjustment should be described in methods (paragraph starting at line 174).

**Response:** We agreed with the reviewer’s suggestions and included the covariates description in the method section. As follow:

Age (<20 years; 20-34 years; ≥35 years), Skin color (white; mixed-race; others), Education level (elementary school; high school; college degree), Marital status (no partner; with partner), Family income (up to 1 MW; 1-3 MW; >3 MW), ABEP class (A and B; C, D and E), Planned pregnancy (no; yes), Prenatal care (public setor; private sector), Abortion (no; yes), Number of childbirths (primiparous; multiparous).

(please see the Methods section, lines 203 - 209).

***Results***
1) Odds ratio for LBW was similar regardless the PC level, since confidence intervals included 1 in the four analysis conducted. However, authors suggest that they were different in text (line 187). Consider rewriting it.

**Response:** We understand the reviewer’s comment, however we have considered that the crude magnitude of associations for both levels could suggest real associations between inadequate PC in level-3 (OR=1.62) and level-4 (OR=1.84). Although the 95% confidence intervals dis not show a statistical significance for level-3 (0.97 – 2.72) and level-4 (0.99 – 3.44), the 95%CI were borderline for both levels. Therefore, the lack of statistical significance probably occurred because our sample size did not provide enough statistical power to the detect an association that could be real. Thus, in this case, statistical significance only tells us about the likely of random error. If our sample size was greater, such an association would have been statistically significant.

Also, after adjusting by confounding variables, statistically significant associations were observed for both levels (please see table-3).

However, we recognize that the absence of statistical significance in the crude analyzes was not mentioned in the text. Therefore, we rewrote the text as follow:

“Despite a borderline 95% confidence interval, women classified with intermediate/inadequate PC were 62% more likely to have LBW child (95% CI: 0.97–2.72) than those with adequate plus/adequate PC, for level-3 criterion. Similarly, for level-4 criterion, women with intermediate/inadequate PC were 84% more likely to have LBW child (95% CI: 0.99–3.44) than women with adequate plus/adequate PC, despite the borderline 95% CI”.

(please see the Results section, lines 240-245).

2) The same occurs with the incidence of preterm birth; only women with inadequate level 2 were at higher risk for preterm birth, but not those in group 4.

**Response:** Similarly, concerning to preterm birth, the 95% CI for level-4 association was borderline (1.00-3.29), while the magnitude of association was 1.79. Rothman et al (2008, p.179-197), affirm that 95%CI only inform about the likely of random error, that is it inform about the sample size and precision simultaneously. Thus, considering that the 95%CI is very sensitive to the sample size, the lack of statistical significance is not an evidence of absence of association. This is particularly true when we face a borderline 95%CI, meaning that statistical significance could be probably reached with the increase of the sample size.

Nevertheless, we recognize that we have not comment in the text about the borderline 95%CI. Thus, we included such an information in the texto, as follow:

“Despite a borderline 95%CI, in the Level-4, women with intermediate/inadequate PC presented 79% higher risk of preterm birth than women with adequate PC quality (OR = 1.79; 95% CI: 1.00–3.29)”

(please see the Results section, lines 250-252).

Rothman, KJ; Greenland, S & Lash TL. Modern Epidemiology. Ed. Lippincot Williams & Wilkins, 2008 (p. 1798-197).

3) Many cells of table 3 are empty, so I cannot evaluate it.

**Response:** We changed the model of the table so that it can be better understood.

Conclusions are not based on the results authors provided.

**Response:** We understand the reviewer’s comment and modified the discussion section. “Despite all the advances to improve maternal and child health, especially with regard to access to prenatal care. Therefore, adequate and adjusted methods for evaluating the quality of the services offered are essential to identify failures and their impact on reducing of outcomes that can lead to infant death.

Thus, in Rio Branco, a direct association was observed between low-quality PC and increased risk of LBW, preterm birth, and vertical transmission of syphilis/HIV/hepatitis. In this way, it is necessary to reduce the inequalities that still exist in low-income countries. The results demonstrate the importance of prenatal care and used a new indicator for PC quality and proved to be a strong candidate to assess the relationship between the quality of PC and unfavorable outcomes at labor and childbirth.”
