# Supplementary material for: Effect of prenatal care quality on the risk of low birth weight, preterm birth and vertical transmission of HIV, syphilis, and hepatitis
Source: PLOS Glob Public Health. 2023 Mar 29;3(3):e0001716. doi: 10.1371/journal.pgph.0001716 (PMC10058091; doi:10.1371/journal.pgph.0001716)
Supplement: Attachment — Submitted filename: Response to Reviewers (2).docx [file pgph.0001716.s002.docx]

**Aguiar *et al*. "Effect of prenatal care quality on the risk of low birth weight, preterm birth and vertical transmission of HIV, syphilis, and hepatitis." (manuscript PGPH-D-22-00381)**

November 06^rd^ 2022, Rio Branco, Acre

Dear editor of the *PLOS Global Public Health*,

We are grateful for your e-mail dated November 06^st^ 2022 concerning the manuscript entitled “Effect of prenatal care quality on the risk of low birth weight, preterm birth and vertical transmission of HIV, syphilis, and hepatitis” (PGPH-D-22-00381).

We greatly appreciate all the contributions from the reviewers to improve the quality and clarity of our manuscript. We have carefully followed reviewers’ comments, and our point-by-point response is provided below, indicating how and where (line numbers) changes were proceeded. Changes are highlighted in red in the text.

Sincerely,

Débora Aguiar

(on behalf of all the co-authors)

RESPONSE TO REVIEWER #1

Thanks for the work you've done. It's has important findings, especially for poor resource settings in a large country, like Brazil. Despite its importance, your work would benefit from some amendments in the text. Please, consider my comments below.

***Abstract***

Line 35: “Thus, this article to evaluate the”. There’s a missing word here, and could it be: ‘this article aims to evaluate’. Or any other that fits here.

**Response:** We app apologize for the mistake, and thank you for the opportunity to revise the text. Thus, we have modified the text as follow: “This article aims to evaluate the effect of the quality of prenatal care process based on Kotelchuck index combined with consultation contents on adverse birth outcomes in a cohort of postpartum women who attended maternity hospitals in Brazilian western Amazon, city of Rio Branco, state of Acre, Brazil, in 2015”.

(Please, see lines 35-38).

Line 43: “>80.0 - 109% expected consultations for GA according to the Kotelchuck Index”. Didn’t understand these figures. What 109% represents?

**Response:** The kotelchuch index assesses the number of prenatal consultations, based on the month of initiation, classifying the adequacy of initiation as follows: 1 and 2 months, 3 and 4 months, 5 and 6 months, 7 to 9 months;

The number of consultations expected by the Brazilian Ministry of Health is: 6 (37 weeks or more), 5 (33/36 weeks), 4 (29/32 weeks), 3 (28/24 weeks), 2 (<24 weeks).

According to Kotelchuck et al (1994), when a woman presents the proportion of consultations observed above the number of consultations expected by gestational age, the final measure combining these two dimensions (#consultations and the gestational age of beginning prenatal care consultation) would fall into one of the two following category:

***More than adequate***: beginning of prenatal care before or during the 4th month and 110.0% of consultations or more (> 7 consultations) in relation to what was expected for that gestational age;

***Adequate***: start before or during the 4th month and 80.0% to 109.0% of consultations (5 to 6 consultations).

Therefore, presenting “>80.0 - 109% expected consultations for GA according to the Kotelchuck Index” means an ***adequate prenatal care***, according to Kotelchuck *index*.

However, we recognize that the number of consultations recommended is missing from the text. Thus, we have included the number of consultations for each Kotelchuck category in METHODS.

(Please, see lines 166-168).

REFERENCES:

KOTELCHUCK, M. An Evaluation of the Kessner Adequacy of Prenatal Care Index and a Proposed Adequacy of Prenatal Care Utilization Index. v. 84, n. AM J Public Health, p. 1914–1920, 1994.

Line 49: You stated ‘level 4’ of adequacy. Maybe you could give information on the other levels or simply omit this information.

**Response:** We appreciate the suggestion and omit this information in the abstract, specifically. However, this information are explained in the Method section.

Lines 55-59: Please, just give information for findings statistically significant.

**Response:** We appreciate the reviewer’s suggestion, and modified the Abstract as follow “Overall cohort, the outcomes incidences were 8.8% for LBW, 9.2% for preterm birth, and 1.1% for vertical transmission (syphilis/HIV/hepatitis). Crude and adjusted OR showed that inadequate PC increased the risk statistically significant of LBW (ORcrude: 1.84; 95%CI: 0.99-3.44; ORadjusted: 1.87; 95%CI: 1.00–3.52), and preterm birth (ORcrude: 1.79; 95%CI: 1.00-3.29; ORadjusted: 3.98; 95%CI: 1.40–11.29)”.

(Please, see the Method section, lines 53-58).

***Introduction***
Line 64-66: I think it’s important to provide an average rate for under 5y mortality for these countries.

**Response:** We comprehend the reviewer’s suggestion and addition this information considered the Estimates of mortality among children under age 5 by UNICEF region “Mortality of children under 5 years of age remains a major public health challenge in low- and middle-income countries, such as Colombia, Mexico, and Brazil^,^ equivalent in Latin America and Caribbean to 16/1000 live births (LB) in 2020¹”.

(Please, see the Introduction section, lines 65-67).

**REFERENCE:**

UN. Levels & Trends in Child Mortality (Report 2020). New York: Inter-agency Group for Child Mortality Estimation; 2020.

***Methods***

Line 134: What SUS means? I think this is the first time it appears on the text.

**Response:** The SUS is the Brazilian Universal Health System, which is the public health system in the country, also known as “Sistema Único de Saúde” (SUS).

We recognize that this term is not clear in the text. Thus, we proceeded the changes as follow: “Rio Branco city holds only two maternity hospitals, one public and one private that also serves the Brazilian public health system (SUS - Sistema Único de Saúde) in a complementary way”.

(Please, see the Method section, lines 133-135)

Lines 156-57: Were the serological tests performed by the study team or information was retrieved from hospital records? If performed by the study team, you must provide details about the procedures for each test.

**Response:** The serological tests performed was retrieved from hospital records. However, we recognize that such an information is not clearly stated in the text. Thus, we proceeded the changes in the text as follow:

“… and vertical transmission of HIV/hepatitis/syphilis (diagnosed by mother-and-child anti-HIV/hepatitis/syphilis serological tests, retrieved from hospital records)”.

(Please, see the Method section, lines 156-158)

Lines 169-173: By reading the description of the method, it looks like that if a women attended more than the expected number of antenatal care appointments, she would be classified as over 110% of the necessary appointments. Is that right? I still think that the description of the method is a bit tough to follow. Would be possible to convert the % into absolute numbers, like instead of using 110%, use XX ANC appointments?

**Response:** We comprehend the reviewer’s suggestion, but as we mention above, according to the Kotelchuck index (1994) when a woman presents the proportion of consultations observed above the number of consultations expected by gestational age, the final measure combining these two dimensions (number of consultations and the gestational age of beginning prenatal care consultation) would fall into one of the two following category:

***More than adequate***: beginning of prenatal care before or during the 4th month and 110.0% of consultations or more (> 7 consultations) in relation to what was expected for that gestational age;

***Adequate***: start before or during the 4th month and 80.0% to 109.0% of consultations (5 to 6 consultations).

Such a strategy is important to adjust this index to the prenatal care recommendations of each country, as this index is widely used by diferent countries (KOTELCHUCK, 1994; BLOCH et al., 2009; BEECKMAN et al., 2013; LI et al., 2018; HEAMAN et al., 2019). However, we recognize that the number of consultations recommended is missing from the text. Thus, we have included the number of consultations for each Kotelchuck category.

**REFERENCES:**

BEECKMAN, K. et al. The relationship between antenatal care and preterm birth: the importance of content of care. The European Journal of Public Health, v. 23, n. 3, p. 366–371, 1 jun. 2013

BLOCH, J. R.; DAWLEY, K.; SUPLEE, P. D. Application of the Kessner and Kotelchuck Prenatal Care Adequacy Indices in a Preterm Birth Population. Public Health Nursing, v. 26, n. 5, p. 449–459, set. 2009.

HEAMAN, M. et al. The Association of Inadequate and Intensive Prenatal Care With Maternal, Fetal, and Infant Outcomes: A Population-Based Study in Manitoba, Canada. Journal of Obstetrics and Gynaecology Canada. 2019 Jul;41(7):947–59.

KOTELCHUCK, M. An Evaluation of the Kessner Adequacy of Prenatal Care Index and a Proposed Adequacy of Prenatal Care Utilization Index. v. 84, n. AM J Public Health, p. 1914–1920, 1994.

LI, C. et al. Temporal trends of preterm birth in Shenzhen, China: a retrospective study. Reproductive Health, v. 15, n. 1, dez. 2018

Line 205: Please, inform how many years each educational category corresponds.

**Response:** We agreed with the reviewer, and added the number of years for each education category as follow: “Education level (elementary school [8 years]; high school [11 years]; college degree [12 years or more])”.

(Please, see the Method section, lines 208-209)

Line 206: What’s de minimum wage? In Brazilian currency and US dollars by the time the data was collected.

**Response:** We apologize for the mistake. In fact we need to standardize the Brazilian minimum wage according to US dollar. Thus, we have included the correspondence of the Brazilian minimum wage according to US dollar in the text, as follow: **“**Family income, the minimum wage equivalent to R$788 or U$250 in 2015 (up to 1 MW; 1-3 MW; >3 MW)”.

(Please, see the Method section, lines 209-211)

Line 207: What’s ABEP class and what it classifies? Remember, you’re submitting your work to an international journal and most readers are not familiar with the Brazilian system.

**Response:** We appreciate and agree with the suggestion. Thus, we have modified the text and included the explanation of what is the ABEP classification as follow:

“…, the Brazilian Marketing Research Companies Association (ABEP- Associação Brasileira de Empresas de Pesquisa) classification (A and B; C, D and E), which is responsible for establishing the method of the Economic Classification Criteria in Brazil”.

(Please, see the Method section, lines 211-214)

**RESPONSE TO REVIEWER #2**

**Abstract/Title**

1) It is well known that prenatal care quality is associated with worst outcomes for the baby. What is new and interesting in this study are the indicators for prenatal care quality and their ability to predict bad outcomes. The title and the abstract should be based on that strength.

**Response:** We appreciate the reviewer’s suggestion, and we intend modified as follow:

**Title:** Prenatal care quality assessment: Kotelchuck index combined to the consultation contents adjusted by gestational age predicts bad childbirth outcomes?

**Abstract**

Background: Averse birth-outcomes still affect newborns worldwide. Although high-quality prenatal care is the main strategy to prevent these outcomes, the effect of prenatal care based on Kotelchuck index combined with consultation contents is still unclear. **Aim:** to evaluate the effect of the quality of prenatal care process based on Kotelchuck index combined with consultation contents on adverse birth outcomes in a cohort of postpartum women who attended maternity hospitals in Brazilian western Amazon, city of Rio Branco, state of Acre, Brazil, in 2015.

2) Second sentence in the abstract has a typo: “… to evaluate to evaluate…”

**Response:** We apologize for the typo and inform that we have corrected it as follow: **“**This article aims to evaluate the effect of the quality of prenatal care process based on Kotelchuck index combined with consultation contents on adverse..”

(Please, see the Abstract section, lines 35-36).

3) The first sentence in the results (abstract) is not clear: “The incidences were 8.8% for LBW, 9.2% for preterm birth (FOR THE ENTIRE COHORT?), 39.4% and 1,1% (???) for vertical transmission…”

**Response:** We agree with the reviewer, there was also a typo or translation error. Thus, we have corrected the sentence as follow.

“Overall cohort, the outcomes incidences were 8.8% for LBW, 9.2% for preterm birth, and 1.1% for vertical transmission (syphilis/HIV/hepatitis)”.

(Please, see the Abstract section, lines 53-54).

4) The conclusion in the text is much better than in the abstract (again… to conclude that quality o prenatal care is associated with worst outcomes is a weak conclusion.

**Response:** We agreed with the reviewer, and modified the conclusion in the abstract as follow:

“The results draw attention to the importance of quality PC in reducing the risks of LBW, preterm birth, and vertical transmission of syphilis/HIV/hepatitis. Moreover, using this proposed quality prenatal care indicator based on Kotelchuck index combined with consultations contents adjusted by GA may accurately predict unfavorable outcomes”.

(Please, see the Abstract section, lines 58-62).

Introduction
5) The introduction is too long. I suggest reducing at least one paragraph.

**Response:** As requested by the reviewer, we have shorten the last paragraph in the Introduction section, from 158 to 121 words.

“Despite the high PC coverage observed in Northern Brazil^9^, this region presents one of the poorest performance of at least one VDRL and HIV tests, and the highest preterm-birth, and LBW rates in the country^13^. Such a controversy is partly explained by the PC low-quality program regarding the utilization and contents (clinical-obstetric procedures and laboratory tests). Nevertheless, no study evaluated the effect of PC quality in this region, combining the Kotelchuck index with the PC content adjusted by GA, on birth outcomes. Therefore, the present study aimed to evaluate the PC quality process (utilization and content) effect on birth outcomes in a postpartum women cohort served by maternity hospitals in the city of Rio Branco, state of Acre, Brazil, in 2015”.

(Please, see the Abstract section, lines 108-117).

6) The objective at the end of the introductions should reflect the conclusion at the end of the manuscript (as explained before).

**Response:** After the reviewer’s comment we realized that in fact the objective and the conclusion were not completely in tune. Therefore, based on the reviewer’s suggestions, we have clarified the study objective and the text conclusion, as follow:

**Objective.** The present study aimed to evaluate the effect of PC utilization combined with the content adjusted by gestational age on birth outcomes in a postpartum women cohort served by maternity hospitals in the city of Rio Branco, state of Acre, Brazil, in 2015.

**Conclusion**: The results of present study leaded us to conclude that despite the low HIV/Syphilis/Hepatitis vertical transmission incidence observed in Rio Branco, the LBW and preterm birth incidence were higher than high-income countries such as Canada, Brussels, and Belgium, and LMIC such as China. Additionally, the proposed method for assessing PC quality process by combining utilization with the content adjusted by gestational age, was able to predict the effect of intermediate/inadequate PC on LBW risk, even adjusting by maternal age and skin color. Also, the risk of preterm birth among those with intermediate/inadequate PC was almost 4-folds higher than those with adequate plus/adequate PC, even adjusted by marital status, and abortions.

In this sense, reducing the inequalities in low-income countries is necessary, as well as reinforce the importance of high-quality prenatal care and of using an accurate indicator for PC quality assessment that combine utilization and content adjusted by GA. Therefore, adequate and adjusted methods for evaluating the quality of services offered are essential to identify and overcome the flaws/gaps, and their impact on reducing birth bad outcomes.

(Please, see the Abstract section, lines 445-461).

Method

7) The study is well design. I would just include what inadequate, intermediate, adequate, adequate plus means in terms of number of consultations since that´s an important part of the study.

**Response:** We appreciate the reviewer’s comment and agree that the number of consultation of each Kotelchuck index category is missing in the methods section. Therefore, in order to overcome such a gap, we included the number of consultations each Kotelchuck index category as follow:

**“**Adequate plus: beginning before or during the 4th month and 110.0% of consultations (≥7 consultations); Adequate: start before or during the 4th month and 80.0 to 109.0% of consultations (5 or 6 consultations); Intermediate: start before or during the 4th month and 50.0 to 79.0% of consultations (3 or 4 consultations) and Inadequate: start after the 4th month and/or less than 50.0% of consultations (1 or 2 consultations) expected for the gestational age”.

(Please, see the Abstract section, lines 172-176).

Results

8) Is reasonable to group syphilis, HIV and hepatitis as they are not frequent, but it would be interesting to know how many of each did you have in your cohort.

**Response:** We agreed with the reviewer, and included the number of syphilis, HIV and hepatitis cases in the Results section:

“The incidence of vertical transmission (08 cases of syphilis, 02 cases of HIV, and 01 case of hepatitis) was...”.

(Please, see the Results section, lines 242-243).

9) Table 3 should also include vertical transmission (or just exclude this outcome of the study).

**Response:** We comprehend the reviewer’s comment, but in table-3 shows the adjusted values of the logistic regression, and due to the low number of cases of vertical transmission, it was not statistically adequate to proceed the multiple logistic analysis.

Nevertheless, we recognize that the number of vertical transmission cases are missing in the Result section. Thus, we have included the number of syphilis, HIV and hepatitis cases in the Results section, as follow:

“The incidence of vertical transmission (08 cases of syphilis, 02 cases of HIV, and 01 case of hepatitis) was...”.

Discussion

10) Again, the authors should better discuss the association of the indicators with vertical transmission and why they are different than the other outcomes (or just exclude vertical transmission of the outcome).

**Response:** The reviewer’s comment is very precise by the fact that since vertical transmission is one of the evaluated outcomes, it should be addressed in multiple logistic analysis. Once the results were different for this outcome, it should be explained in the discussions section or just excluded from the paper.

According to the scientific literature, vertical transmission, altogether with LBW and preterm birth, is considered one of the main birth outcomes associated to PC quality programs. In this sense, assessing the effect of the PC quality on birth outcomes could not exclude vertical transmission. However, in the 2015 postpartum women cohort of Rio Branco, Acre, there were only a few cases of syphilis, HIV and hepatitis vertical transmission, limiting the multiple analysis for this outcome. However, we recognize that the number of vertical transmission cases are missing in the results, and that such a limitation must be addressed in the limitations paragraph at the discussion section.

Therefore, we have included the number of cases in the Result section (please see the answer to the Question #9), and discussed this issue in the limitation paragraph as follow:

“Lastly, although we have also intended to assess the effect of the PC quality on the syphilis, HIV and hepatitis vertical transmission, the few number of cases found in the studied cohort limited the multiple analysis for this outcome. Thus, studies with greater sample size are necessary to analyze the effect of PC quality on syphilis, HIV and hepatitis vertical transmission in Rio Branco, Acre, since the incidence of this outcome was very low in this area.”

(Please, see the Results section, lines 437-442).
